# Ambient Air Pollution and Risk for Stroke Hospitalization: Impact on Susceptible Groups

**DOI:** 10.3390/toxics10070350

**Published:** 2022-06-25

**Authors:** Chia-Hau Chang, Shih-Hsuan Chen, Peng-Huei Liu, Kuo-Chen Huang, I-Min Chiu, Hsiu-Yung Pan, Fu-Jen Cheng

**Affiliations:** 1 Department of Emergency Medicine, New Taipei Municipal Tucheng Hospital, New Taipei 236, Taiwan; b9505021@cgmh.org.tw; 2 Department of Emergency Medicine, Chang Gung Memorial Hospital, Taoyuan 333, Taiwan; penghuei.liu@gmail.com; 3 Department of Neurology, Kaohsiung Chang Gung Memorial Hospital, Kaohsiung City 83301, Taiwan; b9502054@cgmh.org.tw; 4 College of Medicine, Chang Gung University, Guishan District, Taoyuan City 333, Taiwan; bluescratch7@gmail.com (K.-C.H.); outofray@hotmail.com (I.-M.C.); gettingfat720@gmail.com (H.-Y.P.); 5 Department of Emergency Medicine, Kaohsiung Chang Gung Memorial Hospital, Chang Gung University College of Medicine, Kaohsiung City 833, Taiwan

**Keywords:** particulate matter, air pollution, stroke, hospitalization, susceptible group, PM_2.5_

## Abstract

Stroke is a leading cause of death, and air pollution is associated with stroke hospitalization. However, the susceptibility factors are unclear. Retrospective studies from 2014 to 2018 in Kaohsiung, Taiwan, were analyzed. Adult patients (>17 years) admitted to a medical center with stroke diagnosis were enrolled and patient characteristics and comorbidities were recorded. Air pollutant measurements, including those of particulate matter (PM) with aerodynamic diameters < 10 μm (PM_10_) and < 2.5 μm (PM_2.5_), nitrogen dioxide (NO_2_), and ozone (O_3_), were collected from air quality monitoring stations. During the study period, interquartile range (IQR) increments in PM_2.5_ on lag3 and lag4 were 12.3% (95% CI, 1.1–24.7%) and 11.5% (95% CI, 0.3–23.9%) concerning the risk of stroke hospitalization, respectively. Subgroup analysis revealed that the risk of stroke hospitalization after exposure to PM_2.5_ was greater for those with advanced age (≥80 years, interaction *p* = 0.045) and hypertension (interaction *p* = 0.034), after adjusting for temperature and humidity. A dose-dependent effect of PM_2.5_ on stroke hospitalization was evident. This is one of few studies focusing on the health effects of PM_2.5_ for patients with risk factors of stroke. We found that patients with risk factors, such as advanced age and hypertension, are more susceptible to PM_2.5_ impacts on stroke hospitalization.

## 1. Introduction

Stroke is one of the leading causes of death globally, accounting for approximately 11.6% of total deaths worldwide [1]. The incidence of stroke is increasing, particularly in low- and middle-income countries, which account for two-thirds of all strokes [2]. Stroke is recognized as thromboembolism or vascular hemorrhage of the brain, and previous studies have shown a relationship between air pollution and vascular thromboembolism and coagulation [3,4]. Recently, many epidemiologic studies have demonstrated that particulate matter (PM) with an aerodynamic diameter < 2.5 μm (PM_2.5_) had a greater hazard effect than other air pollution [5,6]. Some studies have revealed that air pollution is positively associated with emergency department (ED) visits for myocardial and cerebral infarction [7,8]. Toxicological studies have also revealed that PM exposure might induce vascular endothelial cell activation, proinflammatory cytokine production, and leukocyte recruitment [9].

Regional and seasonal heterogeneities concerning the health impacts of PM_2.5_ have been apparent. Tian et al. gathered data from 172 Chinese cities and discovered the strongest relationship between PM and stroke hospitalizations in cities with higher temperatures or relative humidity [10]. Regional disparities may be explained by community features, such as the prevalence of air conditioning [11], characteristics of residents [12], and weather conditions [13]. Another possible reason for regional and seasonal heterogeneities is the different constituents of air pollutants as well as different PM components in different regions [14]. The combined effects of different air pollutants may exacerbate health hazards [15]. Patient-level characteristics can also influence air pollution. Xia et al. demonstrated that patients with advanced age were more susceptible to the harmful effects of PM_2.5_ on out-of-hospital cardiac arrest [16]. Pan et al. revealed that patients with more cardiovascular disease risk factors were more susceptible to adverse effects of PM_2.5_ on myocardial infarction [8].

The impact of air pollution on stroke is unclear, especially for patients with different risk factors for stroke. The present study evaluated the effects of short-term exposure to PM_2.5_ and other air pollutants on events of stroke hospitalization. Additionally, the potential triggering effects of PM_2.5_ were explored, especially for those with risk factors.

## 2. Methods

### 2.1. Study Population

This retrospective observational study was conducted between 1 January 2014 and 31 December 2018, in an urban tertiary medical center with over 2500 acute beds and an average of over 73,000 adult ED visits per year. The medical records of non-trauma patients over 17-years-of-age admitted with a principal diagnosis of “stroke” (International Classification of Diseases, tenth revision (ICD-10): I63) were extracted from the hospital administrative database. Two trained emergency physicians reviewed the medical records to ensure that the final diagnosis at discharge was “acute stroke”. Demographic factors of age, sex, address, and ED visit time were obtained from the hospital database. Risk factors for stroke, such as comorbidities, hypertension, diabetes, and cardiac arrhythmia, have also been reported [1]. This study was approved by our hospital’s institutional review board (IRB No. 202100933B0) and performed in accordance with the ethical guidelines of the 1964 Declaration of Helsinki and its later amendments or comparable ethical standards. Informed consent was not required for this study.

### 2.2. Pollutant and Meteorological Data

Air pollution data and meteorological conditions were acquired from 11 air quality monitoring stations established in Kaohsiung City in 1994 by the Taiwanese Environmental Protection Administration. Kaohsiung is a tropical city located in southern Taiwan, and it is about 9 m above sea level. Kaohsiung is the largest commercial harbor and the third largest city in Taiwan, with a population of approximately 2.77 million people. Air pollutant measurements and data collection were performed as previously described [5]. Briefly, four “criteria” pollutants of PM_10_, PM_2.5_, nitrate dioxide (NO_2_), ozone (O_3_), and weather conditions that included temperature and relative humidity were obtained from monitoring stations during the study period. Coarse PM (PM_C_), defined as PM with an aerodynamic diameter of 2.5 to 10 μm, was calculated as the PM_10_ level minus PM_2.5_. The daily mean concentrations of air pollutants and weather conditions were then calculated.

### 2.3. Statistical Analyses

A time-stratified case-crossover study design was used to calculate the health effects of short-term exposure to air pollutants. The study design has been described previously [5,17]. In brief, the case-crossover study design is an alternative type of case-control study in which within-subject comparisons are performed using “case” and “control” periods [18,19]. Time stratification was used to adjust seasonality, long-term trends, and day of the week by selecting a “reference day” on the same day of the week and within the same month as the “case day” [20]. The day a stroke patient presented to the hospital was set as lag 0, the day before the episode was lag 1, and the day before lag 1 was lag 2, the day before lag 2 was lag 3, and so on. Conditional logistic regression was used to estimate the odds ratios (ORs) and 95% confidence intervals (CIs) of air pollutants on stroke hospitalization. Subgroup analyses and interaction *p*-values were also calculated to analyze the impact of season and patient-level characteristics. Temperature and relative humidity were included as confounding factors in the model. Potential nonlinear relationships between air temperature, humidity, and stroke hospitalization were determined using the Akaike information criterion (AIC) [21,22]. The ORs were calculated based on the per interquartile range (IQR) increments for each particulate and gaseous pollutant. The significance criterion was set at *p* < 0.05. All statistical analyses were performed using SAS software version 9.3.

## 3. Results

During the 5-year study period, 3774 adult patients were admitted to the hospital with the impression of stroke. A total of 736 patients were excluded from the analysis because the discharge diagnosis was not acute stroke, such as transient ischemic attack, vasculitis, or neuropathy. A total of 323 patients were excluded because the symptom onset time was unclear. Another 296 patients were excluded because they did not reside in Kaohsiung City. The remaining 2419 patients were included in the analysis. Table 1 shows the demographic characteristics of the 2419 patients, which included 1533 (57.0%) male patients with an average age of 68.0 years. Hypertension (65.5%), diabetes (31.9%), and history of stroke (28.3%) were the most common comorbidities.

Table 2 presents the conditions of air pollutants and weather during the study period in Kaohsiung. The mean levels of PM_2.5_, PM_10_, PM_C_, NO_2_, and O_3_ were 26.5 µg/m^3^, 59.5 µg/m^3^, 33.0 µg/m^3^, 16.2 parts per billion (ppb), and 28.8 ppb, respectively. The levels of meteorological factors and air pollutants in each year during the study period are shown in Supplementary Table S1.

Table 3 summarizes the Spearman correlation coefficients for the air pollutants and weather conditions. PM_2.5_ was highly correlated with PM_C_ (*r* = 0.745, *p* < 0.001), and moderately correlated with NO_2_ (*r* = 0.657, *p* < 0.001) and O_3_ (*r* = 0.416, *p* < 0.001).

Before performing conditional logistic regression, AIC was calculated to evaluate the potential nonlinear relationships between temperature, humidity, and stroke. For temperature, the AIC value for the spline model was 6707.882, which was not better than that for the linear model (AIC = 6707.924, *p* = 0.355). For relative humidity, the linear model (AIC = 6700.989) was better than the spline model (AIC = 6703.702, *p* = 0.007). Consequently, a linear model was applied to the conditional logistic regression model according to AIC values [23].

**Figure 1:**
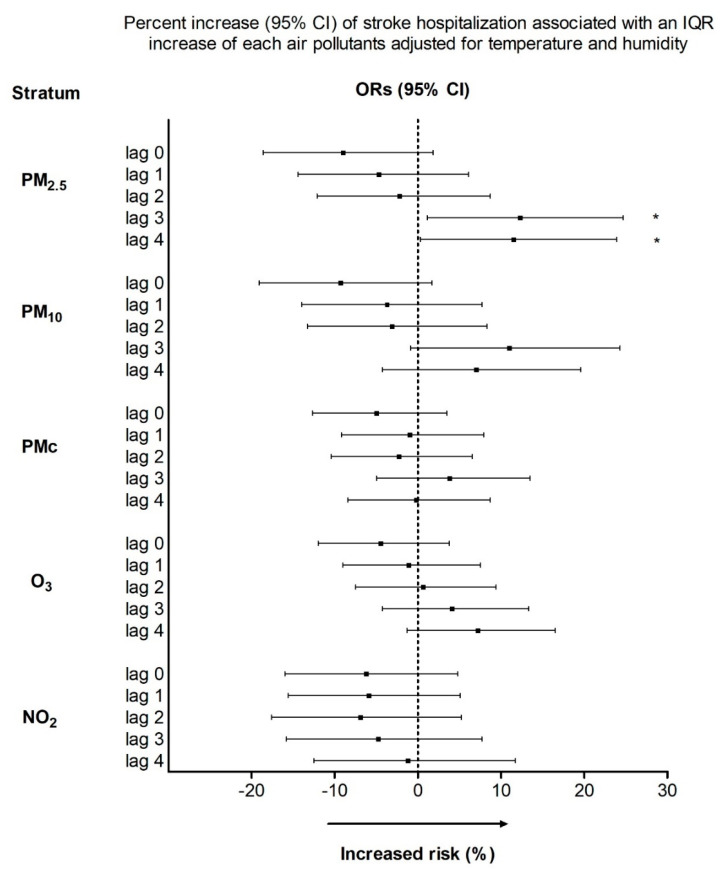
Odds ratios (ORs) and 95% confidence intervals (CIs) for stroke hospitalization associated with IQR increments in each air pollutant.

Figure 1 summarizes the effects of air pollutants on stroke hospitalizations during the study period. An IQR increment in PM_2.5_ on lag3 and lag4 was associated with increments of 12.3% (95% CI, 1.1–24.7%) and 11.5% (95% CI, 0.3–23.9%) concerning the risk of stroke hospitalization, respectively. The impacts of PM_10_, PM_C_, NO_2_, and O_3_ were not statistically significant for lag0 to lag4.

**Figure 2:**
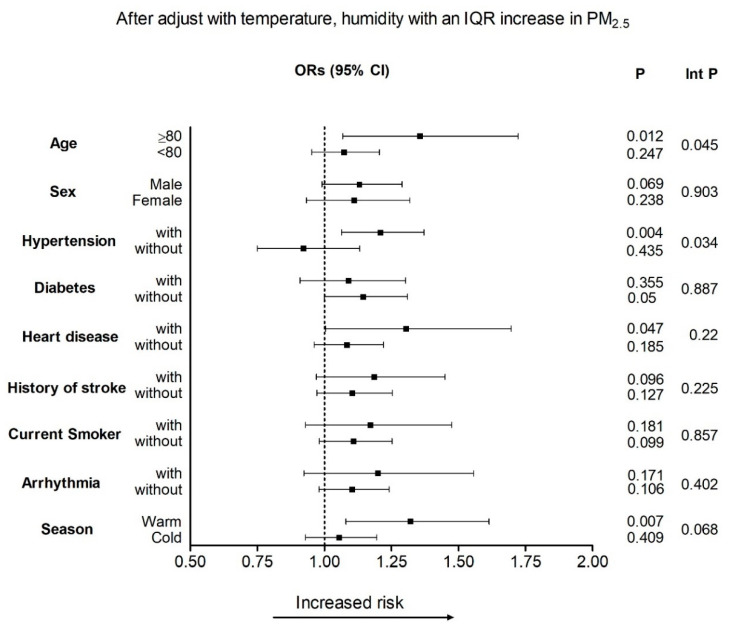
Odds ratios (ORs) for IQR increments in PM_2.5_ on lag3 for different underlying diseases and seasons, after adjusting for temperature and humidity. Int *p* denotes the interaction *p*-value.

Figure 2 shows the results of the stratified analysis to elucidate the effects of PM_2.5_, according to different underlying diseases and seasons on lag3. After adjusting for temperature and humidity, the risk of stroke hospitalization after exposure to PM_2.5_ was greater for those with advanced age (≥80 years, *p* = 0.045) and hypertension (interaction *p* = 0.034).

**Figure 3:**
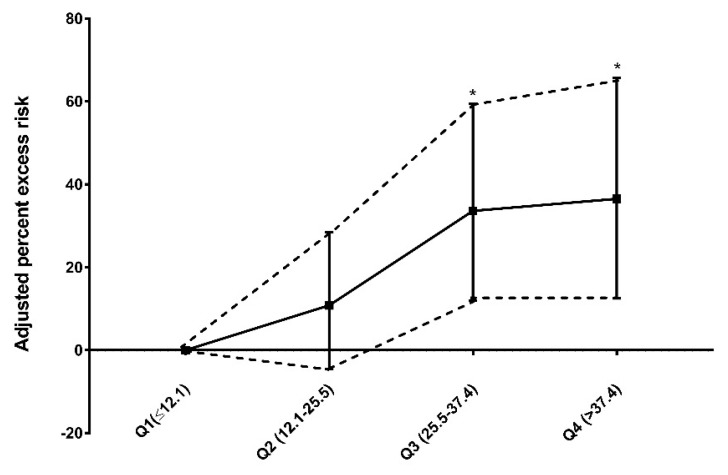
Adjusted risk of stroke according to ambient PM_2.5_ levels. The y-axis represents the percentage of excess risk with 95% confidence intervals. * *p* < 0.01.

Figure 3 shows the exposure–response relationship between the mass of PM_2.5_ and the risk of stroke hospitalization. Compared with lower levels of PM_2.5_ (Q1, ≤12.1 µg/m^3^), elevated levels of PM_2.5_ were significantly associated with an increased risk of stroke hospitalization. Exposure to a Q3 level PM_2.5_ (25.5 to 37.4 µg/m^3^) and Q4 level PM_2.5_ (>37.4 µg/m^3^) was significantly associated with a 33.6% (95% CI, 12.0–59.4%; *p* = 0.001) and 36.5% (95% CI, 12.5–65.7%; *p* = 0.002) increase in the risk of stroke hospitalization, respectively.

## 4. Discussion

The examination of the effects of air pollutants on stroke hospitalization revealed that PM_2.5_ might be significantly associated with the risk of stroke hospitalization in southern Taiwan. Furthermore, patients with advanced age and hypertension were more susceptible to PM_2.5_ during stroke hospitalization.

Air pollution has been associated with many adverse health effects that include asthma, pneumonia, and acute exacerbation of chronic obstructive pulmonary disease [22,24,25]. Previous studies have also revealed the impact of air pollution on cardiovascular diseases, such as myocardial infarction, acute coronary syndrome, and stroke [26,27,28,29]. Concerning stroke, Stafoggia et al. performed a national analysis in Italy, including over 2 million patients who were admitted to the hospital due to cardiovascular diseases. The authors concluded that PM_2.5_ and PM_10_ were associated with the risk of stroke hospitalization [30]. Tian et al. collected data on ischemic stroke from 172 cities in China and found that PM_C_ was positively associated with the risk of ischemic stroke hospitalization, even after adjusting for PM_2.5_ [10]. However, in the latter study, the effect of PM_2.5_ did not achieve statistical significance after adjusting for PM_C_. There are several possible explanations for these disparities. First, the health effects of PM vary regionally and seasonally. For example, Tian et al. observed that the hazard effect of PM_C_ on ischemic stroke was greater in southern China [10], while Ueda et al. reported that PM_2.5_ was associated with daily mortality, especially during spring and autumn [20]. Seasonal and regional differences might lead to differences in PM_2.5_, and different PM_2.5_ components might induce different health hazards [31,32]. Second, different PM components from different combustion sources might induce different health hazards. For example, Pennington et al. revealed that PM_2.5_ from biomass burning and secondary organic carbon was more associated with respiratory disease ED visits, whereas primary coal combustion was more related to cardiovascular disease ED visits [33]. Furthermore, weather conditions and temperatures may interact with air pollution and aggravate health hazards. Huang et al. analyzed 147,624 stroke hospitalizations and concluded that PM_2.5_, PM_C_, and PM_10_ were positively related to the risk of stroke hospitalization on warm days [7]. In the present study, PM_2.5_ exposure was positively associated with stroke hospitalization. The major sources of PM_2.5_ in Kaohsiung were traffic exhaust (18–54%), followed by secondary aerosols and outdoor burning of agriculture wastes [34]. Shah et al. reviewed 94 studies and performed a meta-analysis. The findings demonstrated a positive association between PM_2.5_ concentration and admission or mortality due to stroke [35]. A recent study reviewed epidemiologic studies on PM_2.5_ concentration and cardiovascular disease in China and concluded that PM_2.5_ was positively associated with stroke [36].

Patient-level characteristics may also influence the hazardous effects of air pollution. PM_2.5_ is associated with the risk of out-of-hospital cardiac arrest, especially for those with advanced age, history of heart diseases, diabetes, hypertension, and stroke [16,37,38]. On the other hand, younger age, male sex, and more risk factors for cardiovascular diseases were related to increased susceptibility to PM in myocardial infarction [8,39]. However, subgroup data on the influence of air pollution on stroke are limited. Guan et al. collected data from the China National Stroke Screening Survey and discovered that patients with diabetes are more susceptible to PM_2.5_ [40]. Liu et al. analyzed 59,298 patients with type II diabetes admitted to the hospital due to stroke and found that PM_2.5_ exposure was related to hemorrhagic stroke [41]. However, the interaction *p*-values have not been calculated in previous studies. Noh et al. conducted an epidemiologic study using cumulative average PM_2.5_ to evaluate the chronic health effects of PM_2.5_, and demonstrated that annual mean PM_2.5_ was positively related to the risk of hemorrhagic stroke, especially for those with advanced age (≥65 years) and with obesity [42]. Different responses to PM exposure based on different characteristics have also been mentioned in toxicological studies. Hassanvand et al. examined biomarkers of inflammation after exposure to PM in healthy young adults and the elderly (>65 years of age). They observed elevation of von Willebrand factor, highly sensitive C-reactive protein, and tumor necrosis factor-soluble receptor-II in the elderly group but not in healthy young adults [43]. On the other hand, an animal study revealed that chronic exposure to PM_2.5_ alone induced mild hepatic fibrosis and the hazard effect was augmented in mice fed a high-fat diet [44]. This study also supports different health hazards in patients with different characteristics. We evaluated the short-term hazards of PM_2.5_, especially for those with comorbidities. Patients with advanced age and hypertension were more susceptible to PM_2.5_ on stroke hospitalization.

In addition to PM_2.5_, some gaseous pollutants have been associated with the risk of stroke. Tian et al. performed a nationwide study to evaluate the relationship between air pollutants and ischemic stroke. NO_2_ and sulfur dioxide (SO_2_) were positively associated with stroke hospitalization, even after adjusting for the effect of PM_2.5_, especially in cities with higher temperatures. However, the impact of PM_2.5_ was not statistically significant after adjusting for SO_2_ and NO_2_ [45]. Another study collected data from two large urban areas in Ireland, including stroke admissions and air pollution data. NO_2_ and PM_2.5_ were positively related to the risk of stroke hospitalization only in winter [46]. A review article analyzed the effect of air pollutants on stroke hospitalization or mortality and concluded the hazard effects of PM_2.5_, PM_10_, NO_2_, and SO_2_ on stroke hospitalization and mortality [35]. However, the effects of NO_2_ and O_3_ were not statistically significant in the present study. There are several possible reasons for this finding. First, the health effects of gaseous pollutants may interact with air pollutants and PM_2.5_. Xiao et al. demonstrated the combined effect of NO_2_, SO_2_, and O_3_ on pediatric asthma, as well as the combined effect of PM_2.5_ and O_3_ on pediatric bronchitis [15]. Second, weather conditions may also influence the hazardous effect of gaseous pollutants. A previous study indicated that NO_2_ was associated with the risk of pediatric pneumonia ED visits, and the health effect was greater on warm days [47]. Future studies should include more regions with different climatic conditions to clarify the association between gaseous pollutants and stroke.

## 5. Conclusions

PM_2.5_ may play an important role in the risk of stroke hospitalization in Kaohsiung, Taiwan. Patients with risk factors for stroke, such as advanced age and hypertension, are more susceptible to PM_2.5_ on stroke hospitalization. Besides, the influence of PM_10_, PM_C_, O_3_, and NO_2_ was not statistically significant. Thus, emission limits for PM_2.5_ should be more stringent, especially for those with risk factors.

The present study has some limitations. First, the study was conducted in a single city within a tropical area. Thus, the results may not be applicable to other cities with different climatic conditions. Second, this study was unable to track individual exposures and the use of protective equipment, such as air purifiers and face masks. Thus, individual exposures and outdoor air quality might be different. Third, the time the patients spent outside and the time they moved to other areas could not be recorded in this study. Thus, bias might have occurred.

## Figures and Tables

**Table 1:** Demographic characteristics of patients.

All	Number = 2419	%
Age	68.0 ± 13.0	
Male sex	1533	63.4
Hypertension	1585	65.5
Diabetes	772	31.9
Heart disease	339	13.9
Old stroke	684	28.3
Current smoker	503	20.8
Arrythmia	380	15.7
Warm season	1223	50.6

**Table 2:** Summary statistics for meteorological factors and air pollutants during the study period in Kaohsiung.

	Minimum	Percentiles			Maximum	Mean ± SD
		25%	50%	75%		
PM_2.5_ (µg/m^3^)	1.6	12.1	25.5	37.4	113.8	26.5 ± 15.9
PM_10_ (µg/m^3^)	14.6	35.0	57.2	79.0	176.5	59.5 ± 27.1
PM_C_ (µg/m^3^)	8.2	22.4	30.2	41.8	91.8	33.0 ± 13.1
NO_2_ (ppb)	5.0	10.8	15.3	21.0	187.4	16.2 ± 7.4
O_3_ (ppb)	4.8	19.4	27.7	36.6	187.2	28.8 ± 12.2
Temperature (°C)	7.1	22.8	26.6	29.1	32.1	25.6 ± 4.2
Humidity (%)	35.3	70.0	73.7	77.3	95.9	73.8 ± 6.8

SD, standard deviation.

**Table 3:** Pearson’s correlation coefficients for air pollutants and weather conditions during the study period.

	PM_2.5_	PMc	NO_2_	O_3_	Temperature	Humidity
PM2.5	1.000	0.745	0.657	0.416	−0.647	−0.336
PM_C_		1.000	0.549	0.368	−0.525	−0.402
NO_2_			1.000	0.118	−0.662	−0.177
O_3_				1.000	−0.045	−0.385
Temperature					1.000	0.226
Humidity						1.000

## Data Availability

Data can be made available upon reasonable request.

## Supplementary Materials

The following are available online at https://www.mdpi.com/article/10.3390/toxics10070350/s1, Table S1: Summary statistics for meteorological factors and air pollutants in each year during the study period in Kaohsiung.
