# Supplementary material for: Ambient Air Pollution and Risk for Stroke Hospitalization: Impact on Susceptible Groups
Source: Toxics. 2022 Jun 25;10(7):350. doi: 10.3390/toxics10070350 (PMC9324267; doi:10.3390/toxics10070350)
Supplement: Supplementary file 1 [file toxics-10-00350-s001.zip › toxics-1766254-supplementary.pdf]

Supplementary Table S1 Summary statistics for meteorological factors and air pollutants in each year during the study period in Kaohsiung

Year	$PM_{2.5}$(μg/m³)	PM_{10}(μg/m³)	PM_C (μg/m³)	NO_2 (ppb)	O_3 (ppb)	Temperature (°C)	Humidity (%)
**2014**	29.9±18.3	65.2±30.4	35.3±13.7	17.5±6.7	29.8±12.1	25.2±4.4	73.3±6.3
**2015**	24.7±16.2	62.1±28.5	37.3±16.2	16.5±10.9	28.8±13.5	25.7±3.9	73.2±6.6
**2016**	25.6±15.7	54.5±24.4	28.9±10.3	16.3±6.3	25.9±11.7	25.5±4.4	74.3±6.8
**2017**	27.9±14.3	60.4±26.1	32.5±13.7	15.6±6.0	29.1±11.3	25.8±4.0	73.1±6.5
**2018**	24.3±13.7	55.1±24.0	30.9±12.3	14.9±5.4	30.3±11.6	25.6±4.0	74.9±7.4
